# Post-silicon nano-electronic device and its application in brain-inspired chips

**DOI:** 10.3389/fnbot.2022.948386

**Published:** 2022-07-27

**Authors:** Yi Lv, Houpeng Chen, Qian Wang, Xi Li, Chenchen Xie, Zhitang Song

**Affiliations:** ^1^State Key Laboratory of Functional Materials for Informatics, Laboratory of Nanotechnology, Shanghai Institute of Microsystem and Information Technology, Chinese Academy of Sciences, Shanghai, China; ^2^University of Chinese Academy of Sciences, Beijing, China; ^3^Shanghai Technology Development and Entrepreneurship Platform for Neuromorphic and AI SoC, Shanghai, China; ^4^Shanghai Nanotechnology Promotion Center, Shanghai, China

**Keywords:** brain-inspired chips, post-silicon nano-electronic device, phase change memory, resistive memory, synapse, neuron

## Abstract

As information technology is moving toward the era of big data, the traditional Von-Neumann architecture shows limitations in performance. The field of computing has already struggled with the latency and bandwidth required to access memory (“the memory wall”) and energy dissipation (“the power wall”). These challenging issues, such as “the memory bottleneck,” call for significant research investments to develop a new architecture for the next generation of computing systems. Brain-inspired computing is a new computing architecture providing a method of high energy efficiency and high real-time performance for artificial intelligence computing. Brain-inspired neural network system is based on neuron and synapse. The memristive device has been proposed as an artificial synapse for creating neuromorphic computer applications. In this study, post-silicon nano-electronic device and its application in brain-inspired chips are surveyed. First, we introduce the development of neural networks and review the current typical brain-inspired chips, including brain-inspired chips dominated by analog circuit and brain-inspired chips of the full-digital circuit, leading to the design of brain-inspired chips based on post-silicon nano-electronic device. Then, through the analysis of N kinds of post-silicon nano-electronic devices, the research progress of constructing brain-inspired chips using post-silicon nano-electronic device is expounded. Lastly, the future of building brain-inspired chips based on post-silicon nano-electronic device has been prospected.

## Introduction

**Figure 1:**
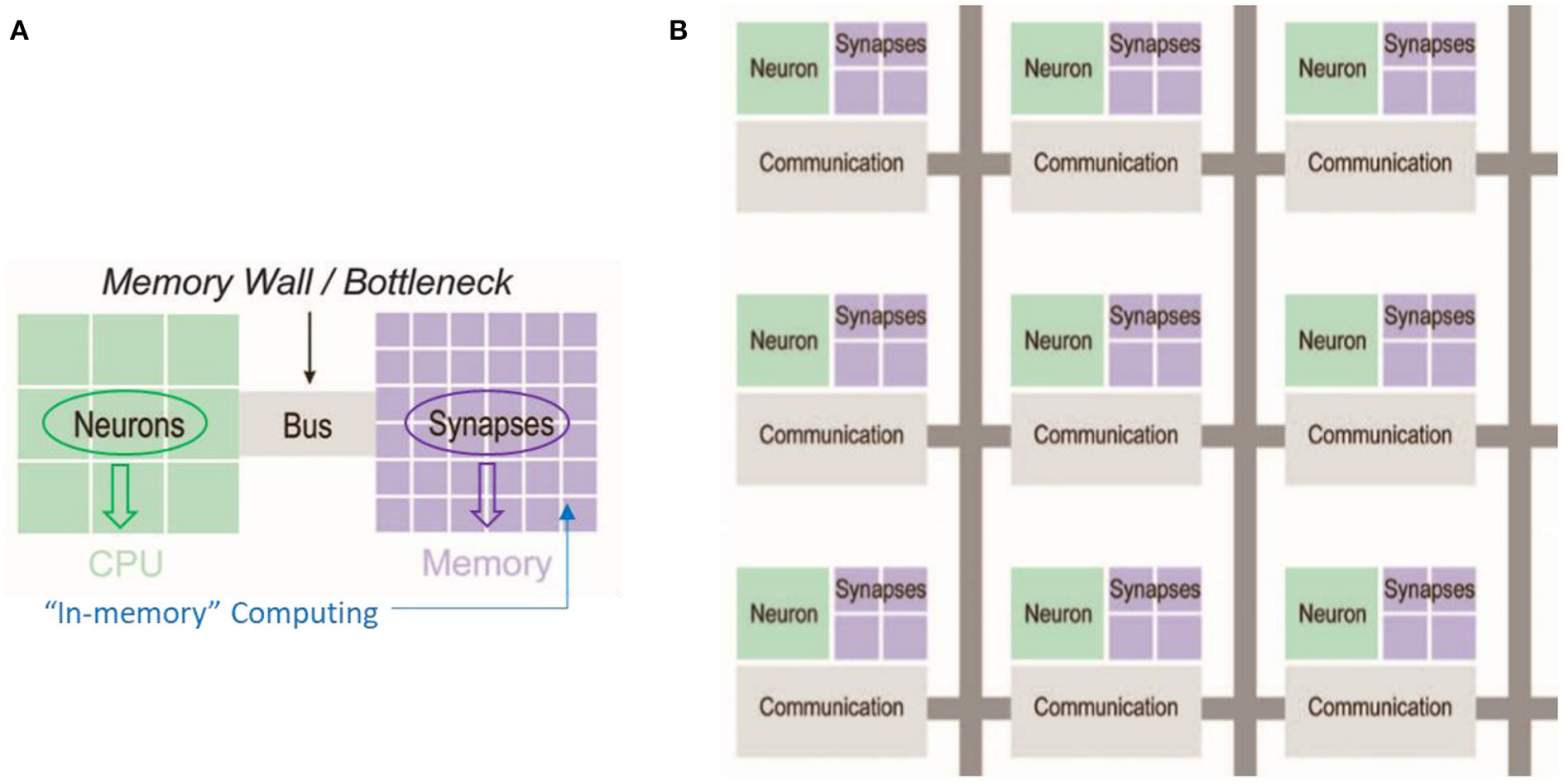
**(A)** traditional Von-Neumann architecture **(B)** Brain-inspired architecture (Burr et al., 2015; Silver et al., 2016).

With the rapid development of big data, the Internet of Things, 5G communication technology, and deep learning algorithms, the amount of data has increased exponentially. The huge amount of data poses a lot of challenges to the storage, processing, and transfer of data. Despite the continuous improvement of computer performance, due to the sharp increase in the amount of computation, there is still a difference of nearly 5 orders of magnitude in the Von-Neumann architecture based on the separation of traditional storage and computation compared with the human brain (Schuller et al., 2015). The traditional Von-Neumann system adopts the separate structure of data storage and data processing. For the data communication process between the computing unit and storage unit, the data processing will produce a lot of loss and latency, which forms a “Von-Neumann bottleneck.” This problem is increasingly highlighted by the fact that CPU speed and memory capacity are growing much faster than the data traffic on both parties (Sun K. X. et al., 2021). This performance mismatch between the storage unit and the computing unit leads to a large delay in the reading of data and in the storage process of the data, that is, the “storage wall” problem. In the case of massive data, it is increasingly overwhelmed. Therefore, it is necessary to explore a new memory architecture based on the human brain structure that achieves low-power consumption, low latency, and space-time information processing capabilities to complete the direct communication of information. Figure 1 shows the traditional Von-Neumann architecture and the new brain-inspired chip architecture (Burr et al., 2015; Silver et al., 2016).

Brain-inspired chips, as the name suggests, are chips that simulate the way the brain works, which is based on the human brain neuron structure and the way of human brain perception and cognition. The chip is designed with the human brain neuron structure to improve the computing power and achieve complete anthropomorphism. Brain-inspired chips adopt a new architecture that simulates the synaptic transmission structure of the human brain. Many processors are similar to neurons and the communication system is similar to nerve fibers. The computing of each neuron is carried out locally. On the whole, the neurons work in a distributed manner, that is, the overall tasks are divided and each neuron is only responsible for one part of the computing.

Brain-inspired chips are based on the combination of microelectronics technology and new neuromorphic devices. Compared with traditional chips, it has greater advantages in power consumption and learning ability. Traditionally, computer chips are designed according to the Von-Neumann architecture. Storage and computing are separated in space. Every time the computer operates, it needs to reciprocate in the two areas of CPU and memory, which leads to frequent data exchanging in inefficient processing of massive amounts of information. In addition, when the chip is working, most of the electrical energy will be converted into heat energy, resulting in increased power consumption.

Brain-inspired chips will achieve two breakthroughs compared with traditional computing chips: one is to break through the limitations of the traditional “executor” computing paradigm and it is expected to form a new paradigm of “self-service cognition”; the other is to break through the limitations of traditional computer architecture to realize parallel data transmission and distributed processing, which will process massive data in real-time with extremely low-power consumption.

The exploration of brain-inspired chips needs to solve the following three main problems: (1) how to deal with the production capacity of flash memory from all over the world far lower than the growth of big data; (2) how to detect useful data in the face of vast big data; (3) how to rely on artificial intelligence to process big data in two directions— digital accelerators and analog neural networks.

This study first introduces the theory of neural networks and the development of brain-inspired chips. Second, the study focuses on the research progress and application of post-silicon nano-electronic devices. Among them, the application of brain-inspired chips is emphasized. Finally, the research and application prospects of post-silicon nano-electronic device brain-inspired chips have been prospected.

## Neural network theory

**Figure 2:**
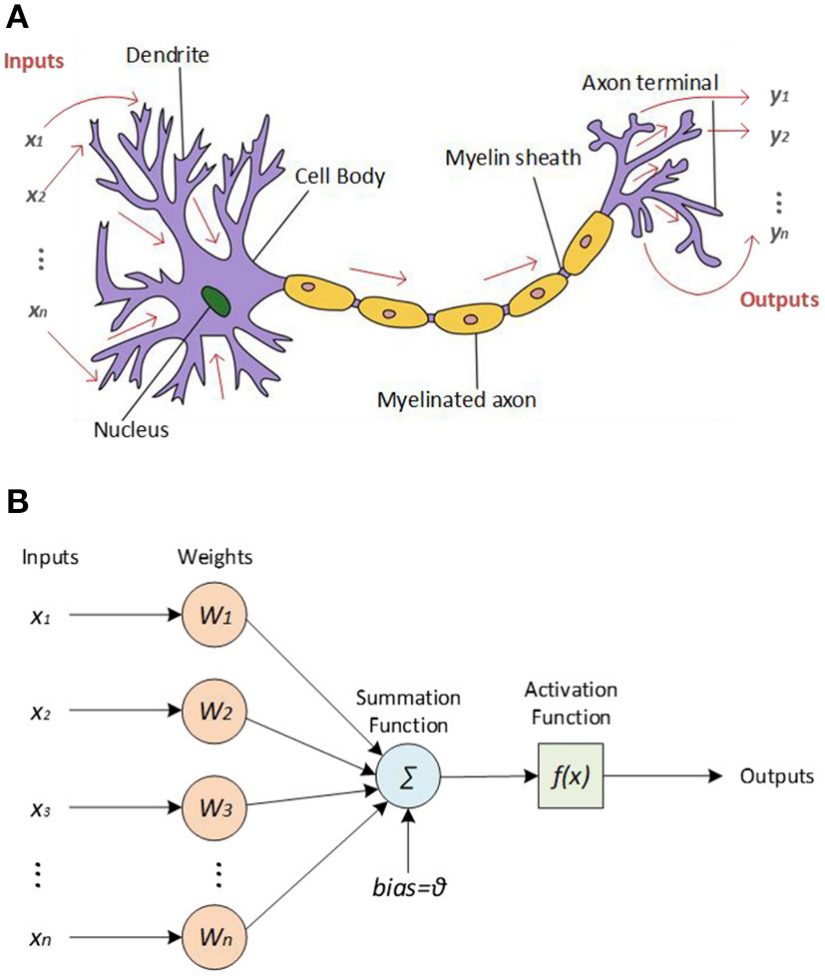
**(A)** Diagram of two neurons' connection structure and synapses **(B)** Schematic diagram of the processing of input signals by neurons in a neural network.

The basic unit structure of the biological neural network is neuron and synapse. As the connection structure between neurons, the synapse is also the medium of data transmission, as shown in Figure 2A. The three basic functions of neurons are to receive data, integrate data, and transmit data. The typical structure of biological neurons consists of the cell body, dendrite, and axon. In a neuronal system, neurons that send signals are called pre-synaptic neurons. Neurons that receive signals are called post-synaptic neurons. The synaptic structure connects pre-synaptic neurons with post-synaptic neurons which transmit data. The weight of synapses reflects the connection strength between units. One of the cores of the biological neural network is the change of synapses for information transmission efficiency, that is, the plasticity of synaptic connections (Thomas, 2013).

Figure 2B shows the processing of input signals by neurons in a neural network. Neurons not only accept input signals but also need to perform data analysis on the input signals. After being stimulated by other neurons, biological neurons do not simply accumulate all the stimuli and output them to the next neuron. Instead, there is a threshold, and only when the neuron receives a stimulus greater than the threshold will it output a distinct stimulus. Neurons in artificial neural networks also have this function. The artificial neuron accumulates all the input signals processed by the artificial synapse. Artificial neurons only output signals when the cumulative signal exceeds a set threshold.

The neural network mainly includes three layers: the input layer, the output layer, and the hidden layer, in which the hidden layers can be expanded. According to the neuron model, neural networks can be divided into two categories: Artificial Neural Networks (ANN) (Hopfield, 1982) and Spiking Neural Networks (SNN) (Maass, 1997).

**Figure 3:**
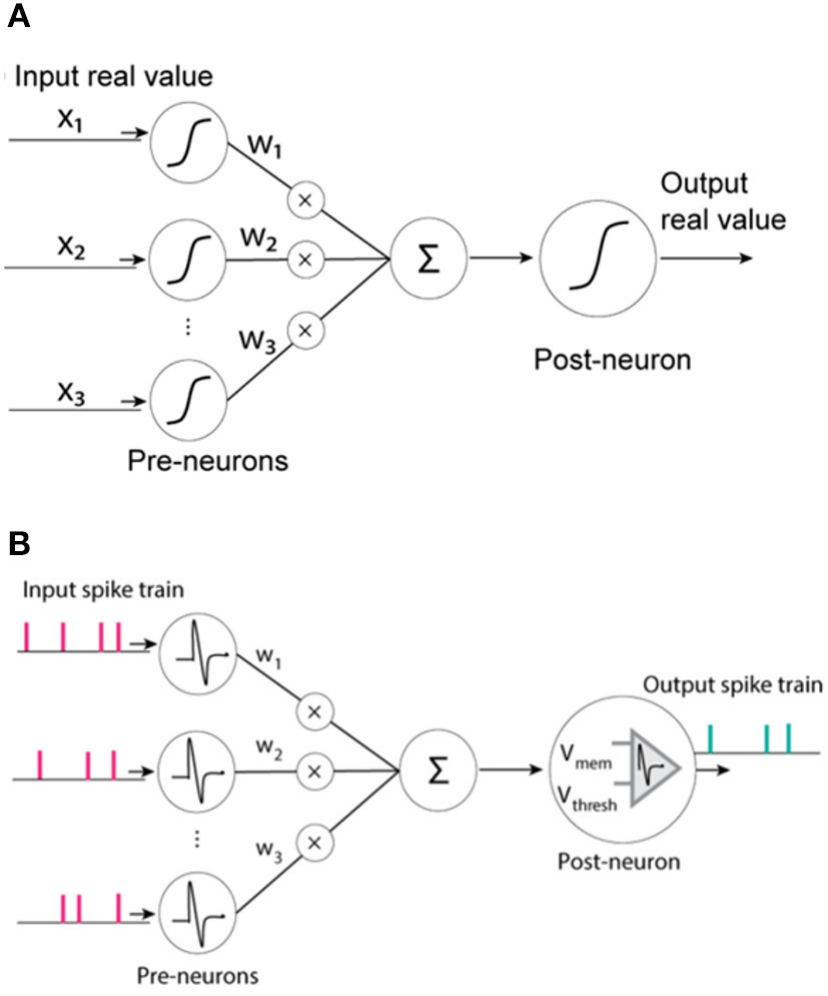
**(A)** Working principle of ANN **(B)** Working principle of SNN (Zhang, 2020).

ANN is an information processing system similar to the human brain nervous system which is established inspired by the structure of the biological neural network. The working principle of the ANN is shown in Figure 3A. When the input signal is received, its intensity is first determined, which is commonly referred to as the weighting process. Then, the combined effect of all input signals needs to be determined, that is, the net input, completing the summation process. Finally, the input is transformed through non-linear function calculation to obtain the corresponding output signal. Among them, the functions of non-linear transformation mainly include the sigmoid function, tanh function, and relu function. The unit structure of ANN is similar to that of the biological neural network, which can complete the learning and cognitive training functions of a biological neural network to a certain extent, usually with the Backpropagation (BP) algorithm (Rumelhart et al., 1986). ANN can learn without supervision, that is, it has the ability of self-learning. The advanced function of realizing the associative storage of the human brain can be accomplished by using its feedback network.

SNN is a neural network computing system based on the spiking neuron model. It is a computing model that is closer to the biological neural network. The working principle of SNN is shown in Figure 3B. The pulse signal is discrete, replacing the continuity of the analog signal in ANN. It is similar to ANN. Because the network also takes the parameters of time information into account, SNN is closer to the biological neuron model. At the same time, the neuron model is also more complicated due to the structure of the pulse signal. From the perspective of the neuron structure in SNN, the input signal will cause the state of the neuron to change, that is, the membrane potential. Only when the membrane potential reaches the threshold potential will the output pulse signal be generated. Among them, Spike timing-dependent plasticity (STDP) algorithm is one of the main learning algorithms of SNN (Fukushima, 1980; Froemke and Dan, 2002).

## Brain-inspired chips

**Figure 4:**
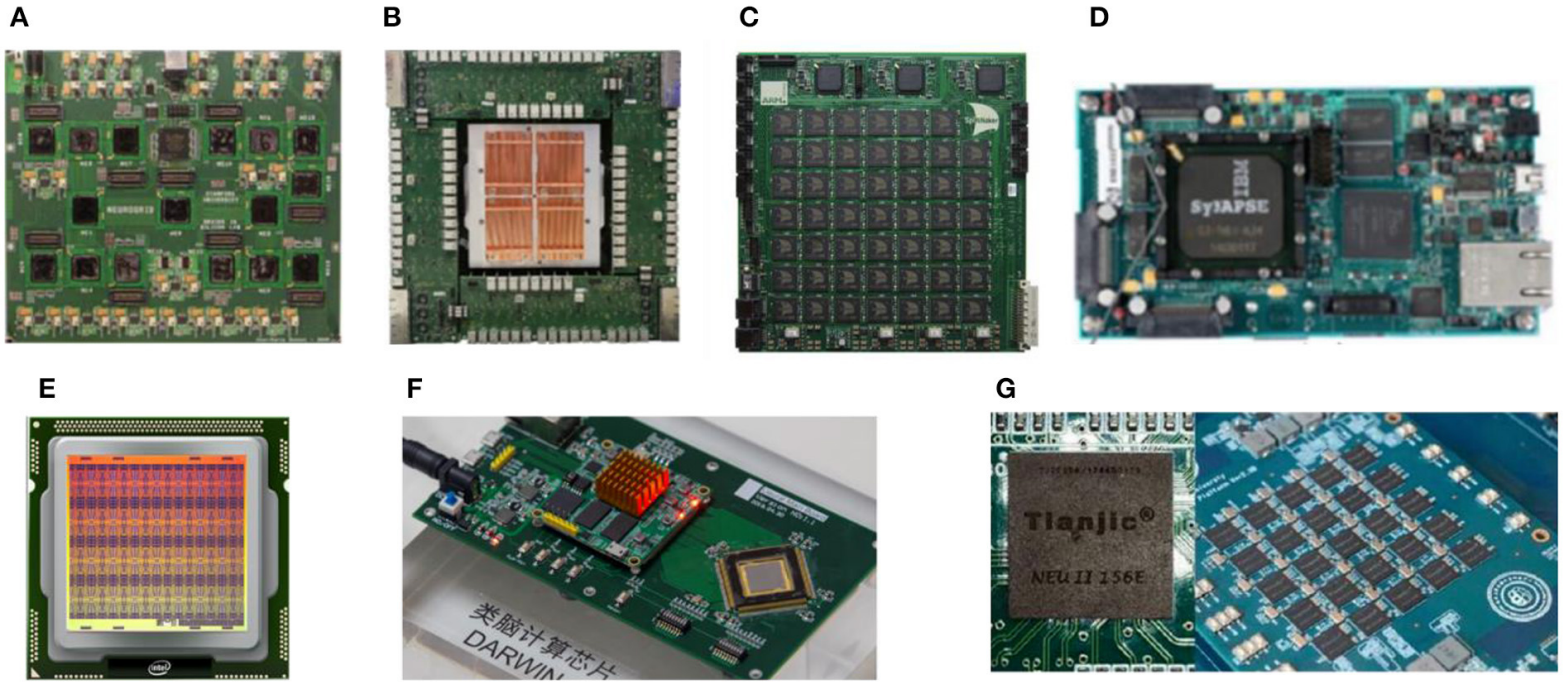
**(A)** The architecture of Neurogrid **(B)** The architecture of BranScaleS **(C)** The architecture of SpiNNaker **(D)** The architecture of TrueNorth **(E)** The architecture of Loihi **(F)** The architecture of Darwin **(G)** The architecture of Tianjic.

At present, brain-inspired chips are mainly divided into brain-inspired chips dominated by analog circuits, brain-inspired chips based on digital circuits, and brain-inspired chips based on post-silicon nano-electronic device. The traditional CMOS technology has been developed to a relatively high degree, and many successful results have been achieved so far. The brain-inspired chip based on a post-silicon nano-electronic device is in the initial stage of exploration and development. At present, the research on brain-inspired chips based on post-silicon nano-electronic device is widely concerned to complete the parallel one-time mapping between input and output. Figure 4 shows the international research status of brain-inspired chips.

### Brain-inspired chips dominated by the analog circuit

As early as the end of the twentieth century and the beginning of the twenty-first century, a series of research works on silicon cochlea and silicon neurons laid the foundation for the design of brain-inspired chips dominated by analog circuits. Among them, the most representative is the Neurogrid chip designed by Stanford University in the United States, which has been established to realize the real-time simulation of the biological brain (Benjamin et al., 2014). It uses the SNN neuron model to realize the kinetic calculation of ion channels and fit complex ion channel models. Its system structure is shown in Figure 4A. Each neuron with a size of 256^*^256 is combined into a neural nucleus, and then 16 neural nuclei are formed into a hierarchical network through a tree topology. Finally, the simulation of a million-level neural network meta-networks is completed.

The BrianScales chip of Heisenberg University in Germany also uses the SNN neuron model to realize the kinetic calculation of ion channels. Its system structure is shown in Figure 4B. A single wafer simulates nearly 200,000 neurons and 49 million synapses. With the cooperation of routing communication circuits, the speed of the entire system is 10,000 times the speed of a biological neural network. However, the power consumption is as high as 1 kW (Davison et al., 2020). The second generation of BrainScaleS adds online learning capabilities and provides an important reference for completing the real-time learning process.

### Brain-inspired chips with full-digital circuit

Because the analog circuit is greatly interfered with by factors such as manufacturing process and environment, the chip does not have advantages in reliability, configurability, scalability, etc. and it is difficult to reproduce the results strictly through simulation, which is not conducive to the research of upper-level algorithms. Therefore, brain-inspired chips based on analog circuits are mainly studied in academia. For the industry, more stable and reliable full-digital circuit brain-inspired chips are preferred (Rast et al., 2010; Benjamin et al., 2014; Merolla et al., 2014; Davies et al., 2018; Davison et al., 2020).

In 2006, the University of Manchester started to develop the SpiNNaker chip, as shown in Figure 4C. The current version is to build an electronic model of the biological brain through 1 million microprocessors from ARM, which can reach 1% of the human brain, achieving the world's first low-power, large-scale digital model of the human brain (Rast et al., 2010), providing a high-performance platform for real-time simulation of large-scale neural networks. The TrueNorth chip released by IBM in 2014 adopts a full-digital circuit, simulating the connection of 1 million neurons and 256 million synapses to complete the neural network function, as shown in Figure 4D, with a very low-power consumption of 73 mW (Merolla et al., 2014). The function of the chip is to perform inference on pre-trained networks, which can be applied to object detection in images. The Loihi chip released by Intel in 2017 contains 128,000 neurons and 128 million synaptic structures, which realizes the complexity of neural network topology and enables on-chip learning with different learning modes (Davies et al., 2018) as shown in Figure 4E. Loihi 2 was released in 2021, which is an upgraded version of Loihi using a new process. It integrates 1 million neurons, but compared with the first generation, the area is reduced by half, and the processing speed is 10 times that of the first generation.

In 2019, Zhejiang University released a new brain-inspired chip, Darwin II, as shown in Figure 4F (Shen et al., 2015). This chip uses a 55 nm process, and the number of neurons in the entire chip reaches 150,000. Through the cascade of chip systems, a brain-inspired computing system with tens of millions of neurons can be constructed. Tsinghua University released a new artificial intelligence chip Tianjic III (Tianjic) in 2019, as shown in Figure 4G (Pei et al., 2019). The chip adopts multi-core architecture, reconfigurable building blocks, simplified data flow, and hybrid coding. It can not only adapt to machine learning algorithms based on computer science but also easily realize brain-inspired circuits and multiple encodings. Table 1 introduces prevalent brain-inspired chips.

**Table 1:** Prevalent brain-inspired chips.

**Name**	**Type**	**Learning**	**Simulation time**	**Capacity**	**Connection**
Neurogrid	Analog-dominated	No	Real-time	256*256 CMOS	USB *via* FX2
BrainScales	Analog-dominated	No	Slower than real-time	180 K neurons	Ethernet
SpiNNaker	Full-digital	No	Real-time	1% of brain capacity	Ethernet
TrueNorth	Full-digital	No	Faster than real-time	4,096 core per chip	AXI bus to SoC
Loihi	Full-digital	Yes	Faster than real-time	4,096 core per chip	Ethernet, USB
Darwin	Full-digital	No	70 MHz Clock	2,048 neurons per chip	UART to USB
Tianjic	Full-digital	Ni	Real-time	40 k neurons per chip	Not specified

### Brain-inspired chips based on post-silicon nano-electronic device

With the continuous development of Moore's Law, the feature size of transistors is getting closer and closer to their theoretical physical limit. It is difficult to improve the development of the current CMOS process integration technology further. When a brain-inspired chip is integrated on a large scale, the larger the area of the circuit is, the higher the power consumption generated. At the same time, transistors have defects in simulating the dynamic characteristics of neurons and synapses, and their ability to simulate brain-inspired computing needs to be further improved. Therefore, researchers turned their attention to post-silicon nano-electronic devices to realize the design of brain-inspired chips.

## The key of brain-inspired chips - post-silicon nano-electronic device

It is urgent to find a memory, whose working behavior characteristics are similar to those of the brain. Brain-inspired chips consist of a large amount of memory. For a long time, researchers have been looking for and constructing suitable post-silicon nano-electronic devices with memory functions. For example, memristive devices can change the working state of the device through different working mechanisms, which is similar to the role of ion channels contained in the membranes of neurons and synapses in the brain. Some memristive devices can keep working like this all the time. Even if the power is turned off, they will not be lost, just like human memory.

Semiconductor memory can be divided into two categories according to the characteristics of stored information: volatile memory (VM) and non-volatile memory (NVM). Generally speaking, volatile memory means that when the system is powered off—all data stored in the device will be automatically lost. It mainly includes two types: Dynamic Random-Access Memory (DRAM) and Static Random-Access Memory (SRAM).

**Figure 5:**
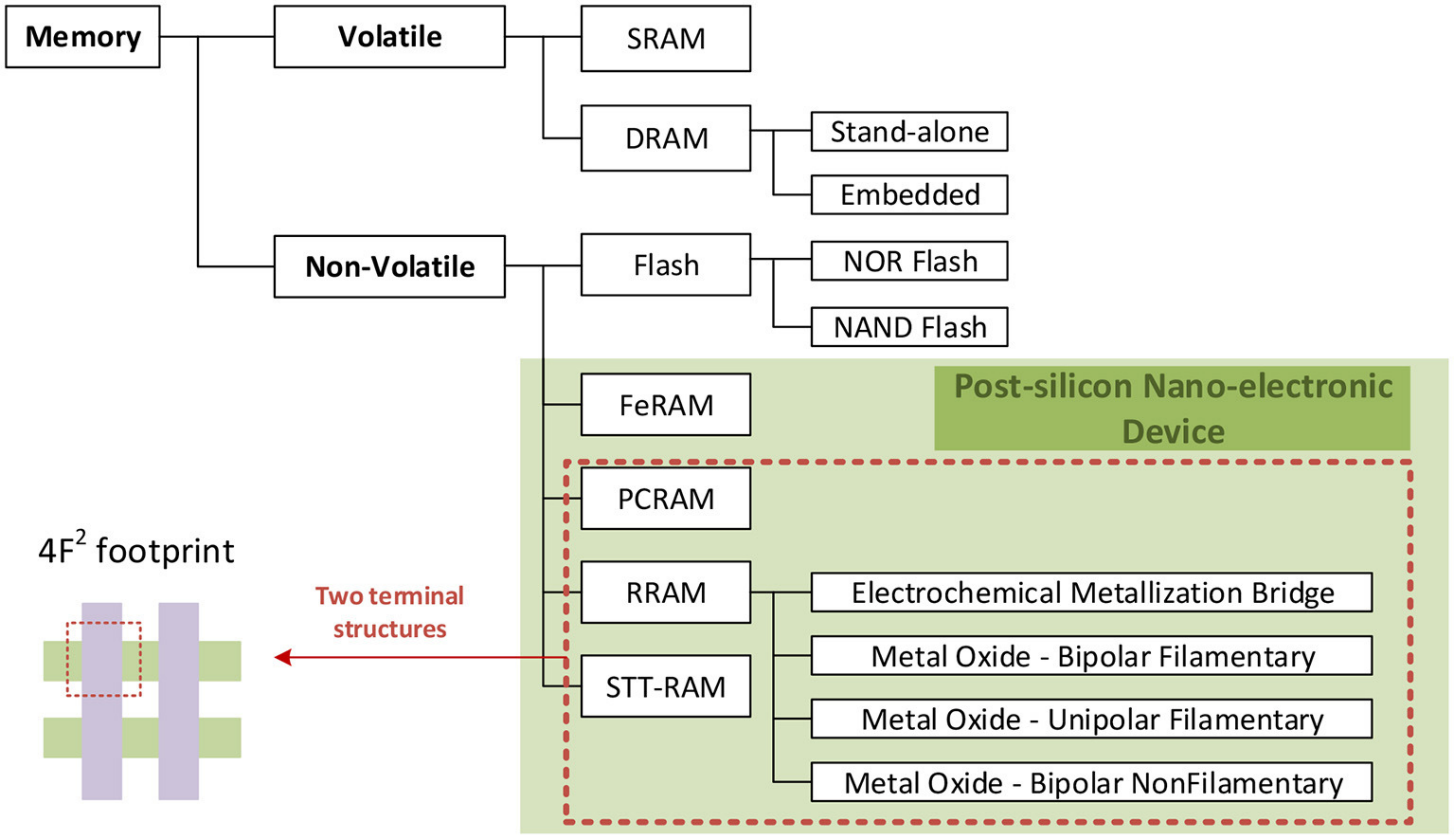
The categories of semiconductor memory.

Non-volatile memory means that when the system is powered off, the data stored in the device will always be retained and will not be lost. It mainly includes new memory and flash memory (Nor Flash memory and Nand Flash memory). Figure 5 shows the main distribution of semiconductor memories on the market today.

In terms of data reading and writing speed, the speed of volatile memory is usually very fast. However, in general, the writing latency of non-volatile memory is high. When the number of writes reaches a certain number, the storage of data will fail because the memory will reach its storage limit. Of course, for an ideal memory, it should have both non-volatile characteristics of data and access speed comparable to SRAM, and no read and write restrictions within a certain range.

Post-silicon nano-electronic device designs and mainstream silicon CMOS processes have different new materials and storage mechanisms. These materials mainly include chalcogenides compounds, transition metal oxides, carbon materials, ferroelectrics, and ferromagnetic metals. Different from the traditional electronic process switching mechanism, they are realized using phase transition, molecular restructuring, quantum mechanical phenomena, and ion reaction. Most non-volatile memories are based on two-terminal switching devices, which are commonly used in high-density memory architectures such as crossbars. In recent years, new storage technologies represented by phase-change random-access memory (PCRAM), resistance random-access memory (RRAM), magnetic random-access memory (MRAM), and ferroelectric random-access memory (FeRAM) have emerged in the field of vision of researchers.

Compared to CMOS technology, which is widely used in chips, post-silicon nano-electronic device-based brain-inspired chips have greater potential in terms of computational density, power efficiency, computational accuracy, and learning ability. In addition, the size of the post-silicon nano-electronic device can be reduced to <2 nm with ultra-high-density integration (Pi et al., 2019). Therefore, post-silicon nano-electronic device technology will be applied to the large-scale manufacturing of brain-inspired chips in the future.

**Figure 6:**
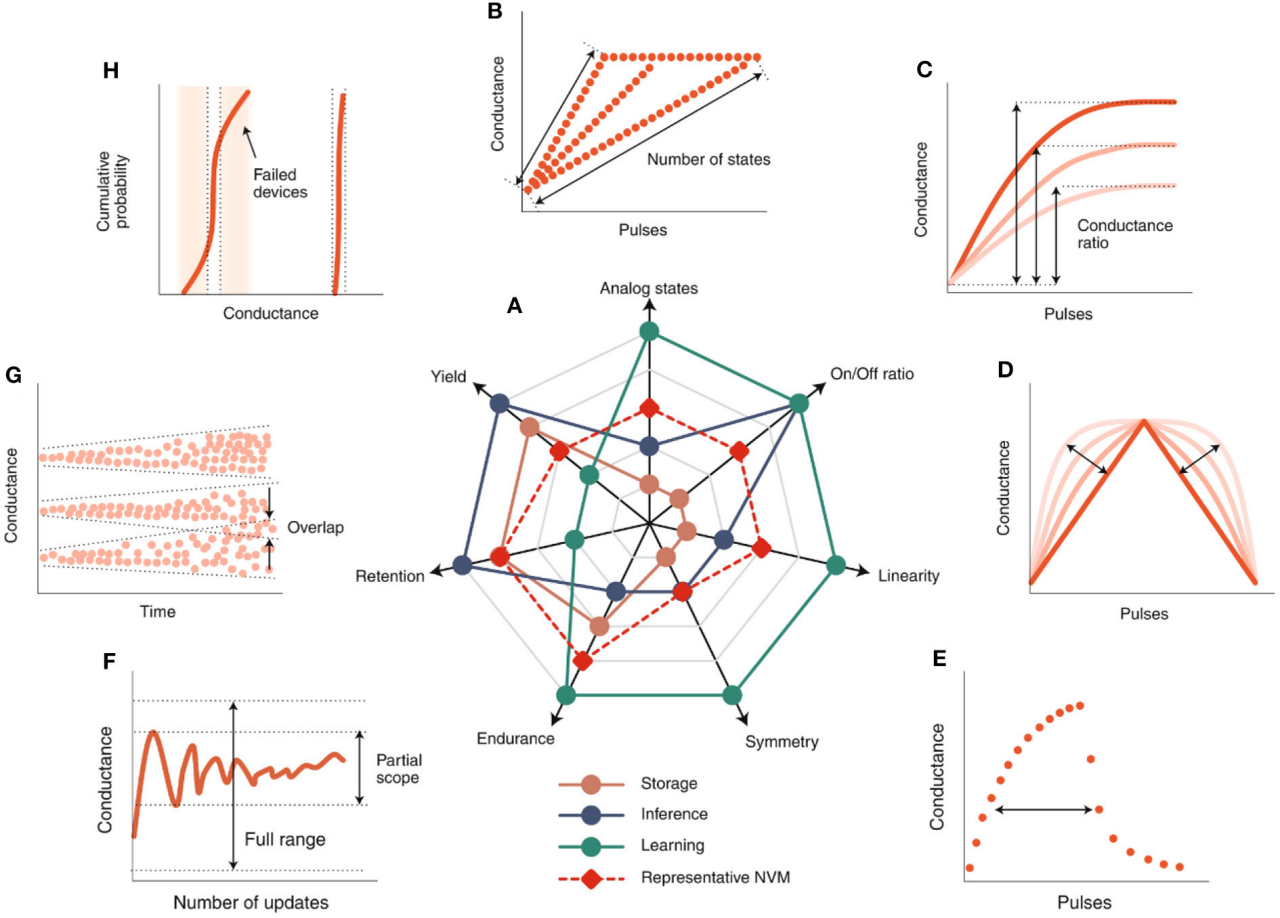
Application-dependent device metric requirements (Zhang W. Q. et al., 2020). **(A)** Ranking of qualitative device requirements for three potential applications and NVM. **(B96-H)**, schematic diagram of computing device requirements: **(B)** simulation state, **(C)** on/off ratio, **(D)** linearity, **(E)** symmetry, **(F)** durability, **(G)** retention rate and **(H)** yield.

The performance requirements of post-silicon nano-electronic device-based brain-inspired chips largely depend on their specific applications. Figure 6A shows the performance requirements for various application scenarios including storage, inference, learning, and typical non-volatile memory. The number of simulated states (Figure 6B) determines the accuracy of weight matching between synapses, and the formation of larger neural networks requires at least 8 resistance states that can be accurately distinguished (Jacob et al., 2017). By optimizing device material selection and circuit design, the current post-silicon nano-electronic device chips can achieve up to 256 resistance states. The dynamic range of switching state transitions is defined as the on/off ratio (Figure 6C) (Wang et al., 2016), which determines the ability to assign the weights in the algorithm to the device conductivity, which in most cases differs from the conductivity of the device in relation to the threshold switch with two resistors. Compared to the high switching ratio, the switching ratio of the multi-resistor post-silicon nano-electronic device is <10. The linearity (Figure 6D) refers to the linearity of the relationship between the conductivity of the device and the number of exciting electric pulses. During the formation of the post-silicon nano-electronic device, the device weights show increasing and decreasing asymmetry (Figure 6E). In the training process, the conductivity update of post-silicon nano-electronic device is usually in the partial scope of the conductivity window, instead of the full range (Figure 6F). After tuning the post-silicon nano-electronic device to different conductance levels, the conductance of the device may change over time, and the two levels may overlap after a period of time (Figure 6G). Failed devices refer to post-silicon nano-electronic device that cannot be tuned to the target conductance Level. (Figure 6H). Based on this, it can be seen that post-silicon nano-electronic device can store weights. According to different application requirements, a suitable new type of post-silicon nano-electronic device can be selected as a memristive device for neural network design (Wang et al., 2022). The memristive device can simulate the function of biological synapses because the sandwich structure of the device unit is similar to nerve synapses (Sun B. et al., 2021c).

### Phase-change memory (PCRAM)

**Figure 7:**
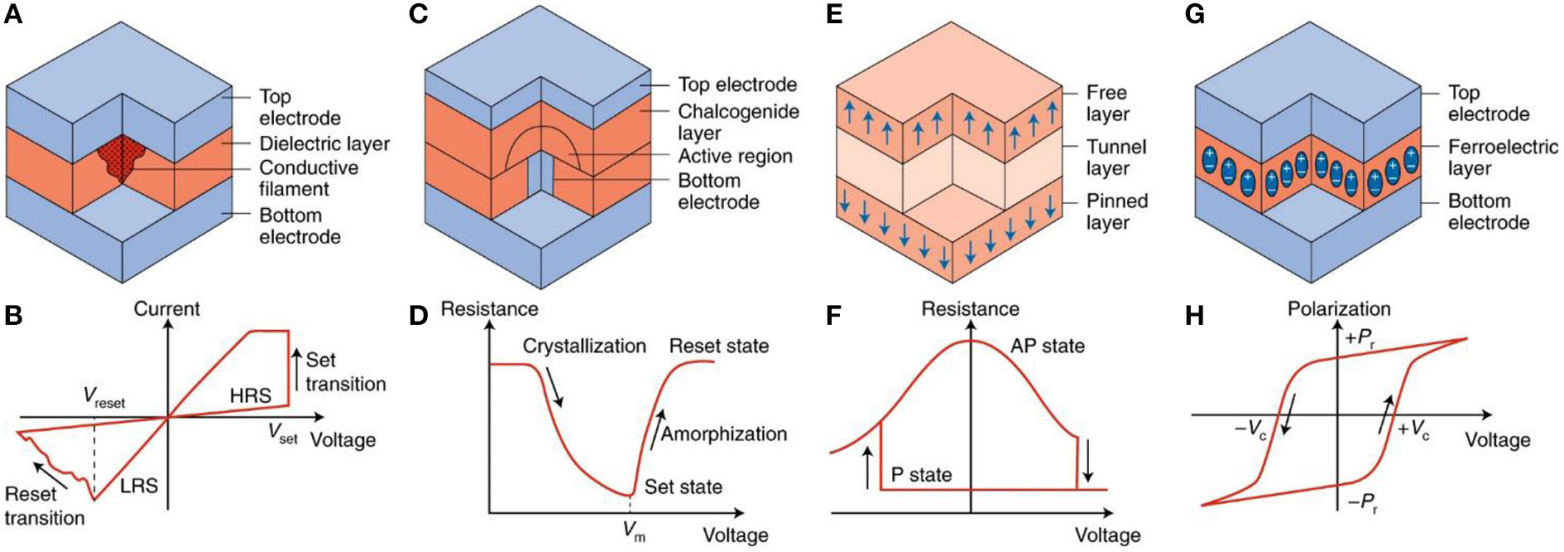
Post-silicon nano-electronic device. **(A)** Conductive filament resistive memory **(B)** corresponding polar current-voltage characteristics **(C)** Phase-change memory **(D)** phase-change memory characteristics **(E)** Spin-transfer torque magnetic random-access memory **(F)** resistance-voltage characteristics of Spin-transfer torque magnetic random-access memory **(G)** ferroelectric random-access memory **(H)** polarization-voltage hysteresis characteristics (Ielmini and Wong, 2018).

PCRAM is a post-silicon nano-electronic device based on GST materials such as Ge_2_Sb_2_Te_5_. According to different device characteristics, the composition of GST material can be further adjusted, as shown in Figure 7C. The resistance change characteristic of PCRAM is shown in Figure 7D. For example, Ge-rich GST (N-type doping) can be used in high-temperature automotive applications for better data retention (Cheng et al., 2012). The switching resistance ratio of phase-change memory is much larger than that of STT-MRAM (in the range of 100 to 1,000 times). Therefore, in principle, Multilevel Cell (MLC) operation is feasible (4 bit/cell has been proposed; Nirschl et al., 2007). A major challenge in PCRAM cell design is the need for a relatively large write current when melting the phase-change material. At present, the structure design trend of phase-change memory is from mushroom type to confined type. The limited type reduces the write current by limiting heat dissipation. Extremely scaled phase-change memory cells using carbon tube electrodes have shown that write currents can reach 1 μA at the 2 nm node (Liang et al., 2011). The resistance drift caused by amorphous relaxation limits the data retention ability of PCRAM, especially for MLC. Therefore, complex circuit compensation schemes are needed. PCRAM has good process compatibility with silicon CMOS technology, regarded as the most mature process technology in the post-silicon nano-electronic device industry (Yu and Chen, 2016).

### Spin-transfer-torque magnetic random-access memory (STT-RAM)

Spin-transfer-torque magnetic random-access memory (STT-RAM) is a kind of memory that stores data by changing the resistance through the magnetoresistance effect of magnetic materials. The basic unit of STT-RAM is a sandwich structure composed of an insulating barrier layer sandwiched between two magneto-resistive materials, which is called a magnetic tunnel junction (MTJ). At the bottom is the fixed layer with fixed polarity, and at the top is the free layer with changeable polarity. The magnetic moment of the free layer is written under the action of the current of the upper and lower wires at the same time. When the magnetic moments of the fixed magnetic layer and the free magnetic layer are parallel in the same direction, the resistance of the magnetic tunnel junction is small. At this time, the device shows a low-resistance state. When the magnetic moments of the fixed magnetic layer and the free magnetic layer are parallel in the opposite direction, electrons are not easy to pass through the magnetic tunnel junction, and the MTJ structure shows a high resistance state, as shown in Figure 7E. The resistance-voltage characteristic of STT-RAM is shown in Figure 7F. STT-RAM stores data “0” and “1” through two different resistive states.

### Resistive random-access memory (RRAM)

RRAM is a kind of post-silicon nano-electronic device that can realize the reversible conversion between high-resistance and low-resistance states under the action of an external electric field based on the resistance of non-conductive material, thus completing the storage of binary data, as shown in Figure 7A. The current96voltage characteristic of RRAM is shown in Figure 7B. According to the different conductive media, it can be divided into two categories: OxRAM (Oxide-RAM), which conducts with oxygen holes, and CBRAM (Conductive Bridge RAM), which conducts with metal ions. The write operation of RRAM includes unipolar and bipolar modes, depending on the oxide as well as the electrode material system. The unipolar mode generally requires larger write currents and has poorer endurance; therefore, the bipolar mode is preferred. A key challenge in the design of the RRAM cell structure is the variability of switching parameters. The significant variation in resistance distribution (perhaps one or two orders of magnitude) presents a challenge to the design of sensitive readout circuits, requiring write-verify techniques to program to the target state, which may at the same time cause delays in MLC operation. RRAM typically has superior process compatibility with mainstream silicon CMOS technologies.

### Ferroelectric random-access memory (FeRAM)

Ferroelectric memory is a post-silicon nano-electronic device with a special process, which is formed by using synthetic lead zirconium titanium (PZT) materials to form memory crystals, as shown in Figure 7G. The polarization-voltage hysteretic characteristic of FeRAM is shown in Figure 7H. When an electric field is applied to a ferrotransistor, the central atom follows the electric field and stops at the low-energy state I. Conversely, when a reverse electric field is applied to the same ferrotransistor, the central atom moves in the crystal along the direction of the electric field and stops in another low-energy state II. A large number of central atoms move and the couples in the crystal unit cell form ferroelectric domains, and the ferroelectric domains form polarized charges under the action of an electric field. The polarization charge formed by the reversal of the ferroelectric domain under the electric field is higher, and the polarization charge formed by the ferroelectric domain without reversal under the electric field is lower. FeRAM combines the advantages of RAM and ROM. Compared with traditional non-volatile memory, FeRAM has the characteristics of high speed, low-power consumption, and long life.

### Comparison of major post-silicon nano-electronic device

The above four major emerging trends are summarized as key strengths and challenges of post-silicon nano-electronic device. PCRAM, RRAM, and MRAM are called resistive memory, while FeRAM is a new memory equivalent to charge memory. Table 2 shows the performance comparison of post-silicon nano-electronic device (Lai and Lowrey, 2001; Song et al., 2008; Sheu et al., 2009; Kim et al., 2011; Tamura et al., 2011; Bez and Cappelletti, 2012; Bez et al., 2013; Zangeneh and Joshi, 2014; Roy et al., 2020; Saxena, 2020). From the table, we can conclude that phase-change memory shows great advantages in terms of high read and write speed, high-density integration, low-energy consumption, low cost, and compatibility with CMOS processes. It can replace the current co-storage structure of DRAM and Flash memory, and its potential in high-speed and high-density storage cannot be underestimated.

**Table 2:** The performance comparison of post-silicon nano-electronic device (Lai and Lowrey, 2001; Song et al., 2008; Sheu et al., 2009; Kim et al., 2011; Tamura et al., 2011; Bez and Cappelletti, 2012; Bez et al., 2013; Zangeneh and Joshi, 2014; Roy et al., 2020; Saxena, 2020).

**Devices**	**MRAM**	**FeRAM**	**PCRAM**	**RRAM**
Non-volatile	Yes	Yes	Yes	Yes
Cell size (F^2^)	8	15–34	4	4
Read latency	30 ns	45 ns	50 ns	8.5 ns
Write/Erase latency	30 ns/30 ns	10 ns/10 ns	10 ns/20 ns	5 ns/5 ns
Endurance	>10^12^	10^14^	>10^12^	10^8^
Write power	High	Low	High	Low
High voltage required (V)	3	2–3	1.5–3	1.5–3
CMOS compatibility	Medium	Medium	Good	Good
Multi-level	No	No	Yes	Yes
3D Xpoint	Yes	Yes	Yes	Yes
Cost	Medium	High	Low	Low

## Research on construction of brain-inspired chips based on post-silicon nano-electronic device

### Synapse

**Figure 8:**
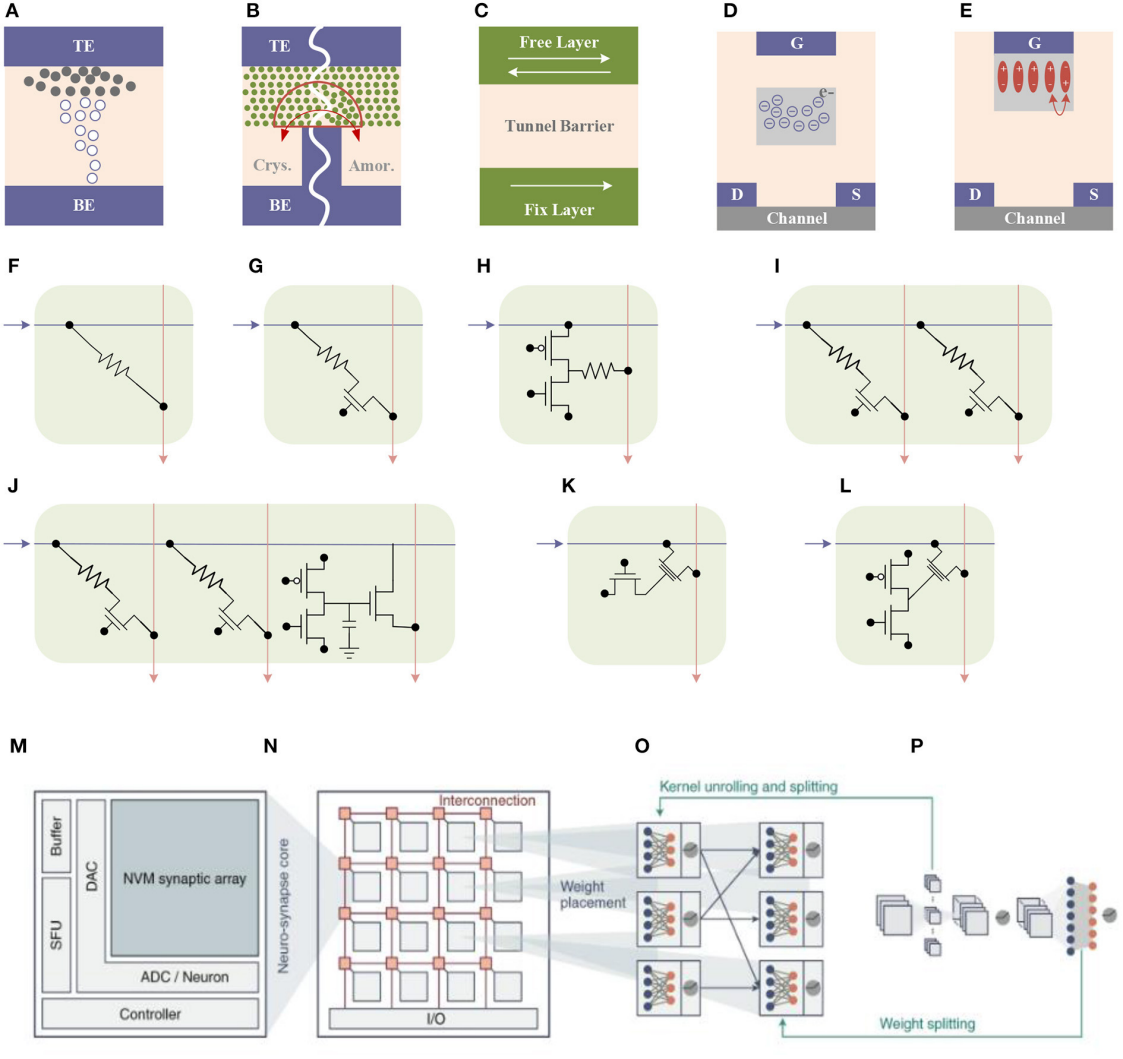
Two-terminal devices: **(A)** RRAM, **(B)** PCM, and **(C)** MRAM. Three-terminal devices: **(D)** flash memory and **(E)** FeFET. Post-silicon nano-electronic device-based cell structure: **(F)** 1R synapse, **(G)** 1T1R synapse, **(H)** 2T1R synapse, **(I)** 2T2R synapse, **(J)** 2T2R+3T1C synapse, **(K)** 1T+1TriR synapse and **(L)** 2T+1TriR synapse. **(M)** neuro-synapse core, **(N)** neuro-synapse core in brain-inspired chips **(O, P)** neural network working process (Zhang W. Q. et al., 2020).

Combined with the design and application of brain-inspired chips, different types of non-volatile memory devices have been proposed. In the application of neural networks, according to the relationship between the adjustment of weight and the reading of weight, these devices can be divided into two categories: two-terminal devices and three-terminal devices. The two-terminal devices mainly include PCRAM, RRAM, and MRAM. Three-terminal devices mainly include flash memory and ferroelectric memory as shown in Figure 8.

#### PCRAM

**Figure 9:**
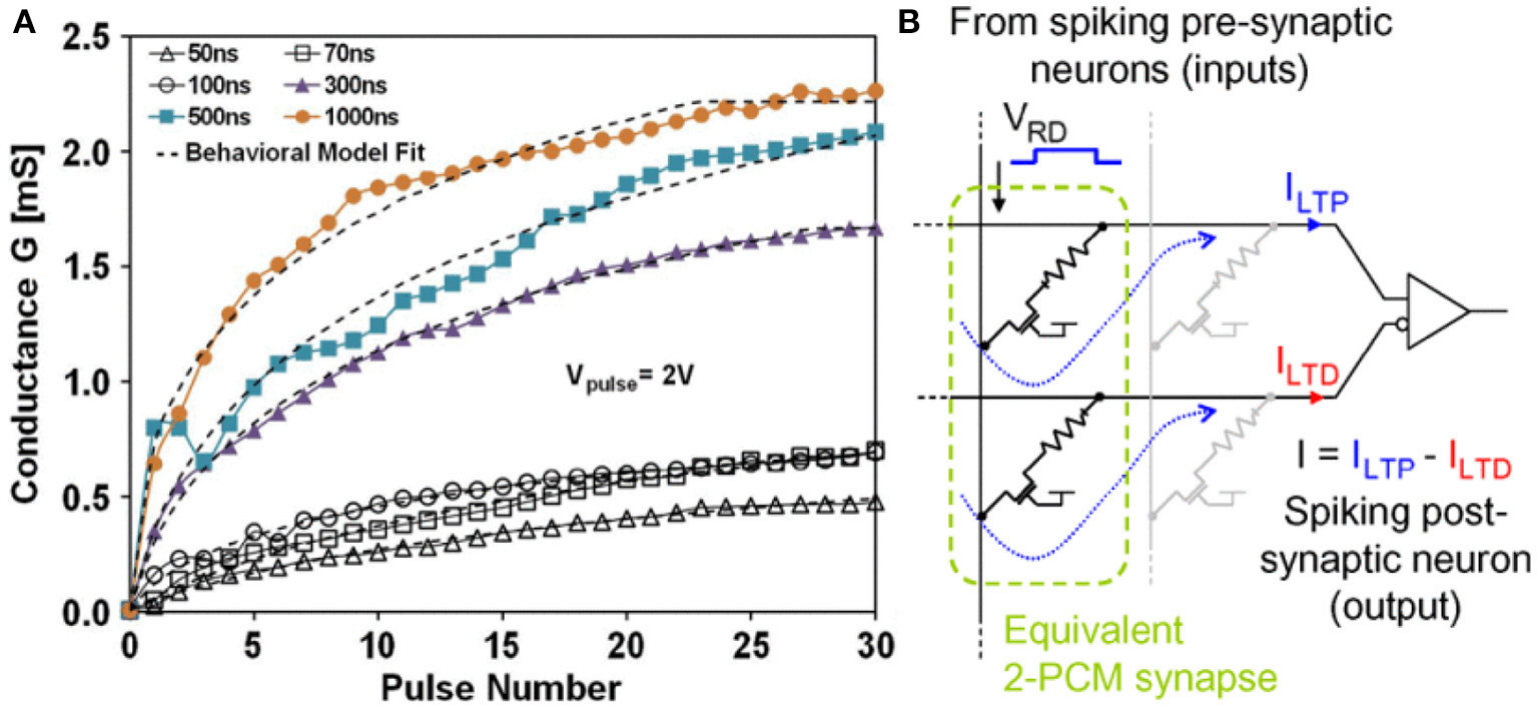
**(A)** Experimental LTP characteristics of Ge_2_Sb_2_Te_5_ (GST) PCM devices. **(B)** 2-PCM synapse principle (Bichler et al., 2012).

Work on a PCM-based device was first proposed in 2012 (Kuzum et al., 2012). By applying a series of incremental excitation pulses to the device, the resistance of the device can change under about 100 resistance states, and under appropriate pulses, the learning rule of spiking-time-dependent plasticity (STDP) can be realized under waveform. Subsequently, different research groups proposed various excitation pulse programming schemes to reduce the complexity and power consumption of PCM-based neuromorphic circuits (Suri et al., 2011; Jackson et al., 2013; Li et al., 2013; Stefano et al., 2016). However, a major challenge of PCM devices is the asymmetry of the resistance switching process, which is mainly because the process of melting the material at a high temperature to form an amorphous state is more difficult to control than the process of recrystallization of its amorphous state. Phase-change memory can achieve multilevel resistance states by the programming pulse. Only two resistance states can be achieved during reset using the same pulse. To this end, Bichler et al. proposed a 2-PCM synapse design to deal with this problem in their work (Bichler et al., 2012), in which one PCM was used as a synaptic potentiation (Long-term potentiation, LTP), and the other was used as a synaptic depression (Long-term depression, LTD). In this design, both PCM devices are partially crystallized. During LTP and LTD, the conductance of the device is increasing. The current through the LTP device plays a positive role and the current through the LTD device plays a negative role. The current through the LTD is subtracted at the output, ultimately resulting in synaptic inhibition, as shown in Figure 9.

#### RRAM

In the early RRAM device design, the artificial synapse device based on HfO_x_ material adopted the one-way reset learning mode (Yu et al., 2013). To make this process smoother, multiple conductive filaments can be formed under the electric field through the design of multilayer oxides implemented in the device. In the RRAM device with an interface mechanism, the resistance changes during the set and reset process are relatively gentle (Park et al., 2012, 2013; Gao et al., 2015b; Wang et al., 2015). In addition, multi-resistance states can also be achieved by regulating the capture and release of interfacial oxygen vacancies (Yang et al., 2017). The resistive switching device exhibited multistate resistance behavior, which enables 2-bit storage capacity in a single device providing a method for logic in-memory and neuromorphic computing (Sun B. et al., 2021b). A memristive device and a hybrid system composed of CMOS neurons and RRAM synapses were experimentally demonstrated to realize essential synaptic functions such as STDP (Jo et al., 2010).

Depending on the application, different excitation pulse programming schemes are applied for online or offline training with RRAM, so the requirements for device characteristics may vary. For example, in the offline training process, the resistance state can be iteratively programmed into the specified target layer by the write-verify method. Since the programming process is one-time, accuracy is more critical than speed in the writing process. Alibart et al. simulated this programming process by firing a series of pulses (Alibart et al., 2011), where pulses with smaller amplitudes approach the state in smaller steps but take longer than pulses with larger amplitudes. Therefore, the use of a pulse train of variable amplitude can approach the desired state in small steps within a reasonable time frame. In the absence of a change in switching state, the pulse amplitude becomes progressively smaller, resulting in smaller steps as the device gets closer to the desired state. However, due to the fluctuation of the device itself, the process of determining the initial pulse value often starts with a small non-disturbing pulse and gradually increases, and the conductance of the device is confirmed by applying the read pulse after the write pulse until the required accuracy is achieved. When using this method, because the initial state is very close to the desired state, the maximum amplitude of the voltage pulse written in the new sequence is smaller than that of the previous sequence, which can ensure that the device is closer to the desired state. For a single Pt/TiO_2−x_/Pt device, this method can adjust the conductance to any expected value in the dynamic range of the device with an error of only 1% (Alibart et al., 2011). For the Ag/a-Si/Pt single device, the tuning accuracy for the low-resistance state is also close to 1%. A similar iterative algorithm has also been demonstrated in H_f_O_x_ devices (Gao et al., 2015a). For online training, since the synaptic weights need to be dynamically trained, the programming speed becomes a more important factor, therefore, smooth conductance adjustment without write verification becomes the preferred solution (Yu, 2018). Some examples of state-of-the-art based on RRAM are given in the literature, all of which show bidirectional graded conductance tuning under the same programming voltage pulse (Mulaosmanovic et al., 2017; Yu, 2018). Although these devices can all reach tens or hundreds of resistive states, there are still non-linearities and asymmetries in the tuning. They used W/MgO/SiO_2_/Mo memristive device as the synapse of speech recognition and completed the hardware implementation of SNN using the improved supervised tempotron algorithm on the TIDIGITS dataset (Al-Shedivat et al., 2015; Wu et al., 2022).

#### FeFET

FeFET synapse devices use a three-terminal structure, which is characterized by decoupling the write and read paths for the resistive state of the device. In FeFET, the programming voltage applied to the gate determines the resistance change of the device. The current is given by the drain-source current read. As mentioned earlier, as a three-terminal device, FeFET is designed for weighted summation as pseudo cross arrays. In terms of physical structure, FeFET is to apply short voltage pulses through the gate through the multi-domain effect in ferroelectric materials, so as to gradually adjust the capacitance of the gate, and finally complete the adjustment of threshold voltage and channel conductance (Oh et al., 2017). Recently, (Jerry et al., 2017) simulated FeFET synaptic devices using a gate-last manufacturing process flow of n-channel FeFETs, whose gates were formed by stacking 10 nm Hf_0.5_Zr_0.5_O_2_ (HZO) materials by atomic deposition and annealed at 600°C to generate multiple ferroelectric domains in HZO nanocrystals. Compared to RRAM devices, FeFETs have advantages in on-off ratio and available program pulse range with less variation in the weight update curve.

### Neuron

Neuromorphic computing systems need to simulate not only synapses, but also neuronal dynamics, including membrane potential maintenance, transient dynamics, and neurotransmission processes (Burr et al., 2016). In human neurons, the maintenance of membrane potential depends on the ion pump and ion channel in the middle of the membrane lipid bilayer. The excitation or inhibition of post-synaptic potentials of neuronal dendrites can change their state. In neurons composed of phase-change memory, the membrane potential is represented by an amorphous state of high resistance, and the firing frequency of phase-change neurons is controlled by the amplitude width and time interval of a series of voltage pulses. Connecting the plasticity of synapses, such neurons can complete complex calculations such as detecting time correlation in parallel data streams.

When a post-silicon nano-electronic device is used to build a neuron, the goal of the device is not the continuity of its conductance state, but rather a cumulative behavior that fires after receiving a certain number of pulses. Since each conductance state of a post-silicon nano-electronic device affects its behavior between accumulation and emission pulses, changes in these conductance states will be the focus of research.

The use of PCM devices to construct neurons was first reported in the work of Ovshinksy and Wright (Wright et al., 2011). In their work, Tuma et al. changed the membrane potential of neural components through phase encoding, and then experimentally proved that neurons based on PCM devices can integrate post-synaptic input signals (Tuma et al., 2016). A system in which both neuron and synaptic devices were implemented using PCM devices was reported by Pantazi et al. (2016). Studies by Averbeck et al. have shown that stochastic behaviors in neuronal dynamics, such as ionic conductance noise and thermal noise-induced chaotic motion of charge carriers, morphological variation between neurons, and other background noise can also affect neuronal signaling. Encoding and transmission play a key role (Averbeck et al., 2006). Therefore, simulating these random behaviors in artificial neurons can achieve many interesting functions (Maass, 2014). The random behavior in the device is due to the inhomogeneity of the thickness of the amorphous region and the internal atomic configuration during melt quenching of different batches of materials, and these random behaviors can lead to multiple integrations of the signal generated by a phase transition in the PCM neuron. The interval is generated between the transmitted signals to facilitate some statistical calculations based on these transmitted signals. At the same time, however, the melt quenching process of PCM device materials, especially the elemental migration therein, limits the device's durability. Likewise, in RRAM devices, large changes in conductance can also result in reduced device durability. Therefore, extending the lifetime of the device requires ensuring that neurons accumulate and fire the number of spiking signals or fabricating the device with high-durability materials.

**Figure 10:**
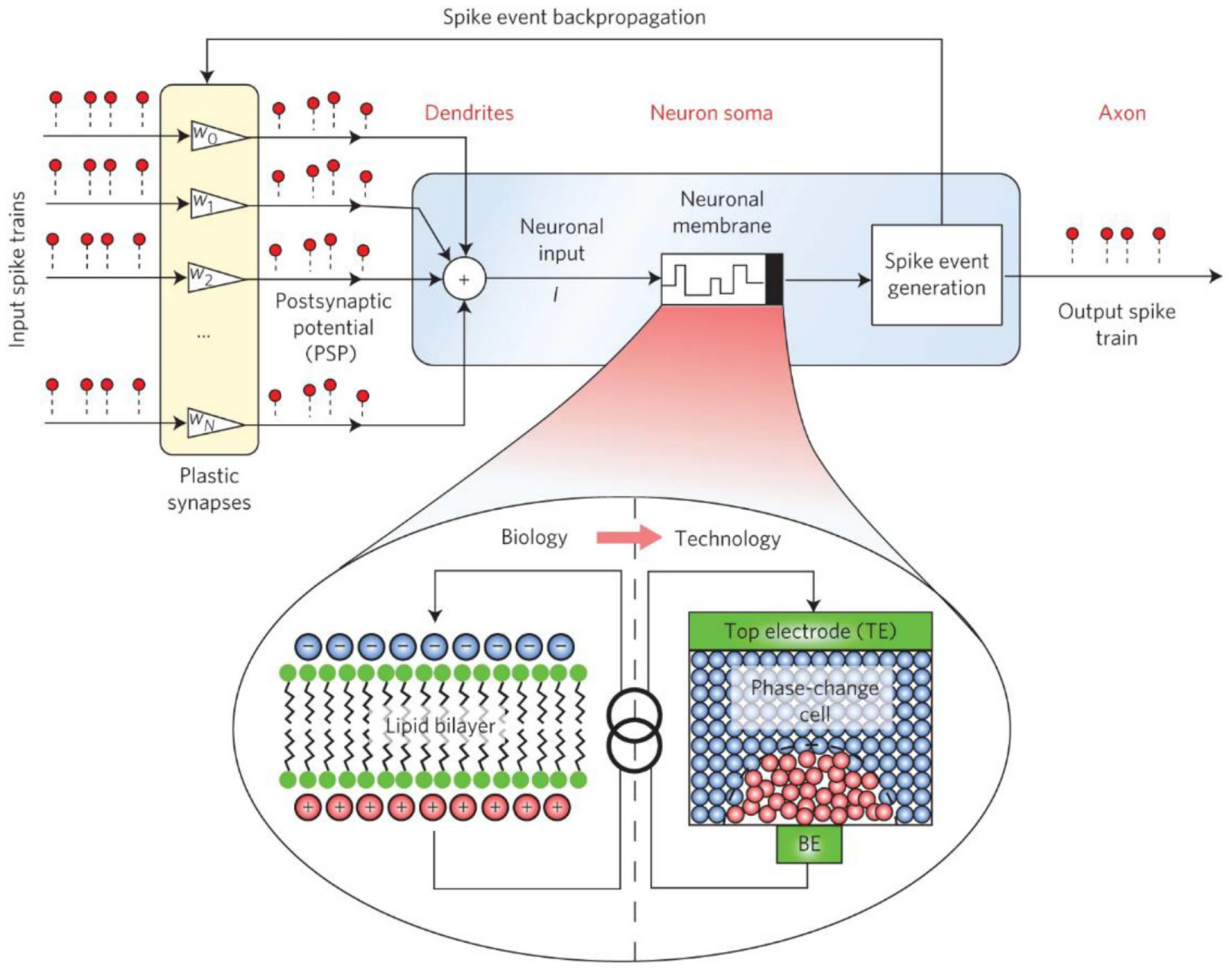
Basic structure diagram of IBM phase-change neuron (Tuma et al., 2016).

Figure 10 (Tuma et al., 2016) shows the basic structure diagram of the IBM phase-change neuron. The synapses consist of phase-change units that are responsible for weighting incoming excitation signals. Multiple excitation signals are input into the synaptic array, and after the signals pass through the synapse, they are input into the phase-change unit that functions as a neuronal membrane (neuronal membrane, which can also be understood as a neuron). When the threshold is reached, the IF event is triggered, and the excitation signal is emitted. The excitation signal is firstly conducted to the outside for further data processing, and at the same time, it is back-propagated for comparison with the previous input excitation signal. For positive delays, synaptic conductance is increased, and for negative delays, synaptic conductance is decreased. These functions of synapses can be achieved with SET and RESET operations. Through the above analysis, it can be found that this system has met the main requirements of the bionic neural network.

Al-Shedivat et al. have proposed to use TiO_x_-based RRAM to construct random artificial neurons (Al-Shedivat et al., 2015). In an RRAM, integrating the input signal of neurons increases the voltage across the capacitive device, that is, increases the membrane potential of neurons, causing the device as a whole to switch to the low-resistance state and the generated increased current is converted into digital by an external circuit signal or analog pulse. Meanwhile, random switching of resistive states in RRAM results in random firing of neurons (Nessler et al., 2013). Jang et al. also implemented a similar principle on a Cu/Ti/Al_2_O_3_-based conductive bridge random-access memory (conductive-bridging RAM; CBRAM) (Jang et al., 2017).

Resistive memory has also been used in the simulation of axonal behavior. The neuron resistor (Neuristor) was first proposed as an analog device for the Hodgkin-Huxley axon (Hodgkin and Huxley, 1952; Crane, 1962), but it could not be mass-produced in the early stages of the concept. Pickett et al. fabricated a neuron resistor composed of two nanoscale Mott memristors based on the Joule heat-driven insulation-conductor phase transition principle (Pickett et al., 2013). This neuron utilizes the dynamic resistance switching behavior of the Mott memristor and the functional similarity between Na^+^ and K^+^ channels in the Hodgkin-Huxley model to make the resistor have all-or-nothing pulse signal gain, periodicity, etc. important neuron features.

Many research works provide more references for the practical application of memristor. A.Chandrasekar et al. studied impulsive synchronization of stochastic memristor-based recurrent neural networks with time delay and concluded that the memristive connection weights have a certain relationship with the stability of the system (Chandrasekar and Rakkiyappan, 2016). Researchers have also done a lot of research on the complete definition of the brain elicitation system and learning mode. The definition of completeness for brain-inspired systems was put forward by Zhang et al. (Zhang Y. et al., 2020), which is composed of Turing-complete software abstract model and a versatile abstract brain-inspired architecture, providing convenience for ensuring the portability of programming language, the completeness of hardware and the feasibility of compilation. By introducing a brain-inspired meta-learning paradigm and a differentiable spike model combining neuronal dynamics and synaptic plasticity, Wu et al. proposed a brain-inspired global-local cooperative learning model. It achieves higher performance than a single learning method (Wu et al., 2020). Associative memory is an important mechanism to describe the process of biological learning and forgetting. It is of great significance to construct neural morphological computing systems and simulate brain-inspired functions. The design and implementation of associative memory circuits have become a research hotspot in the field of artificial neural networks. Pavlov's conditioned reflex experiment is one of the classical cases of associative memory. The implementation of its hardware circuit still has some problems, such as complex circuit design, imperfect function, and unclear process description. Based on this, researchers combined the classical conditional reflection theory and nano-science and technology to study its circuit. Sun et al. put forward a memristive neural network circuit that can realize Pavlov associative memory with time delay achieving learning, forgetting, fast learning, slow forgetting, and time-delay learning (Sun et al., 2020). A memristor-based learning circuit that can realize Pavlov associative memory with dual-mode switching, auditory mode, and visual mode, was designed and verified by Sun et al. (2021a). Sun et al. proposed a memristor-based neural network circuit of emotion congruent memory, which considers various memory and emotion functions, achieving the functions of learning, forgetting, changing speed, and emotion generation (Sun et al., 2021b). Gao et al. experimentally demonstrated the *in situ* learning ability of the sound localization function in a 1K analog memristor array with the proposed multi-threshold-update scheme (Gao et al., 2022), representing a significant advance toward memristor-based auditory localization system with low-energy consumption and high performance.

In 2016, Sengupta et al. proposed a deep spiking neural system based on magnetic tunnel junction (MTJ), which lead to a fully trained deep neural network (DNN) transformed into an SNN on forwarding inference (Sengupta et al., 2016). The input signal of DNN is encoded as a Poisson spike sequence of SNN according to the rate and is regulated by the synaptic weights, resulting in a post-synaptic current flowing through heavy metals under the MTJ device, which causes the switching of the device state in the MTJ device, the probability of which is the distribution is approximated by the DNN sigmoid function, again with a 50% probability of zero input by adding a constant bias current. Stochastic micromagnetic simulations of large-scale deep learning neural network architectures show that SNN forward inference can achieve a test accuracy of up to 97.6% on the MNIST handwritten digit database. Sharad et al. also suggested using lateral spin valves and domain wall magnets (DWMs) as neural components to achieve multiply-accumulate functions (Roy et al., 2013). Initially conceived, this work connects two input magnets with opposite polarities, a stationary magnet, and an output magnet through a metal channel. The transmission of spin torque makes the output magnet switch to a flexible axis parallel to the polarity of the input magnet, which is detected by MTJ.

In a later envision, the device instead uses two magnets with fixed and opposite polarities, which are connected through a DWM device with an integrated MTJ. One magnet is grounded and the other is used to receive the difference between the excitatory and inhibitory currents plus the bias current to center the response of DWM. Such current differences determine the direction of the current flowing through the DWM and the resulting magnetic polarity, which is then induced by the MTJ. Sharad et al. also proposed circuit integration schemes of unipolar and bipolar neurons, as well as device-circuit joint simulation of some common image processing applications. Moon et al. realized pattern recognition neuromorphic systems by combining Mo/PCMO synaptic devices with NbO2 insulator-metal transition neuronal devices, in which the Mo/PCMO devices exhibited excellent performance due to their high activation energy during oxidation reliability (Moon et al., 2015).

## Conclusion

The development of artificial intelligence is highly dependent on massive amounts of data. Meeting the data processing requirements of high-performance machine learning is the most important factor for brain-inspired chips.

This study summarizes the development of brain-inspired and post-silicon nano-electronic device and its applications in brain-inspired chips. The current representative post-silicon nano-electronic device artificial synaptic devices include PCM, RRAM, and FeRAM. In addition, the post-silicon nano-electronic device can also be used to construct neural components. As CMOS technology is approaching its physical limits, post-silicon nano-electronic device-based brain-inspired chips offer a promising path forward.

The brain-inspired system has a broad application prospect in the field of artificial intelligence and cognitive computing because of its low-power consumption and fast parallel computing speed (Sun B. et al., 2021a). The research on brain-inspired chips has made phased progress, but there is still no intelligent system that can approach the human level. In the next period, the research on brain-inspired chips will focus on enhancing the universality of neural computing circuit modules, as well as reducing the difficulty of design and manufacturing. In addition, there is an urgent need to solve the power consumption problem of brain-inspired computing chips, such as exploring ultra-low-power materials and computing structures, to lay a foundation for further improving the performance of brain-inspired chips.

Future device research should focus on implementing simulated post-silicon nano-electronic device with improved performance and exploring more bio-trustworthy properties.

1. Post-silicon nano-electronic device represented by phase-change memory is continuously optimized. In the future, they will continue to improve device performance, develop large-scale integration technology, and realize heterogeneous integration and three-dimensional high-density integration of various neuromorphic devices.

2. Small-scale brain-inspired chip circuits continue to improve in terms of synaptic structure and neuron function. In the future, the collaborative design will be opened to develop large-scale scalable, and versatile post-silicon nano-electronic device-based brain-inspired chips to realize massive data processing.

3. SNN still lacks effective learning algorithms, lacks dedicated hardware platforms, and has few commercial products, which only have theoretical advantages. The research space is relatively large, and the realization of learning algorithms and hardware has broad research prospects.

Brain-inspired chips have propelled the development of brain-inspired supercomputers, giving them extreme computing speeds and massive data processing capabilities. In the future, they can also “cognition” and “thinking,” which will change the traditional working mode of computers.

## Author contributions

YL, HC, and ZS brought up the core concept and architecture of this manuscript. YL, HC, QW, XL, CX, and ZS wrote the article. All authors contributed to the article and approved the submitted version.

## Funding

This work was supported by the National Natural Science Foundation of China (92164302, 61874129, 91964204, 61904186, 61904189, 61874178), 0Strategic Priority Research Program of the Chinese Academy of Sciences (XDB44010200), Science and Technology Council of Shanghai (17DZ2291300, 19JC1416801, 2050112300), by the Youth Innovation Promotion Association CAS under Grant 2022233 and in part by the Shanghai Research and Innovation Functional Program under Grant 17DZ2260900.

## Conflict of interest

The authors declare that the research was conducted in the absence of any commercial or financial relationships that could be construed as a potential conflict of interest.

## Publisher's note

All claims expressed in this article are solely those of the authors and do not necessarily represent those of their affiliated organizations, or those of the publisher, the editors and the reviewers. Any product that may be evaluated in this article, or claim that may be made by its manufacturer, is not guaranteed or endorsed by the publisher.
